# A Rare Case of Optic Nerve Schwannoma: Case Report and Review of the Literature

**DOI:** 10.7759/cureus.265

**Published:** 2015-04-18

**Authors:** Wyatt L Ramey, Stacy J Arnold, Alexander Chiu, Michael Lemole

**Affiliations:** 1 Neurosurgery, University of Arizona; 2 Pathology, University of Arizona; 3 Otolaryngology, University of Arizona; 4 University of Arizona

**Keywords:** endoscopic endonasal, myelination, optic nerve, schwannoma

## Abstract

Background and Importance: Schwannomas are typically benign tumors of the peripheral nervous system that originate from Schwann cells. It is well known that the optic nerves are myelinated by oligodendrocytes since their cell bodies arise centrally within the lateral geniculate nuclei. Because of this basic cellular anatomy, optic schwannomas should theoretically not exist. It is possible, however, these rare lesions stem from small sympathetic fibers that innervate the vasculature surrounding the optic nerve and its sheath.

Clinical Presentation: The patient is a 46-year-old male with a one-year history of progressive right eye blurry vision. To our knowledge, there are only five known reported case of an optic nerve schwannoma. Additionally, because of its medial position relative to the optic nerve and within the orbital apex, it is the first such case to be resected via an endoscopic endonasal approach. The lesion was subtotally resected because of its adherence and continuity with the optic nerve and the patient’s wish to preserve his vision. He was subsequently referred to radiation oncology for external beam radiation therapy.

Conclusion: Herein, we discuss the pertinent clinical findings of this rare lesion and review the literature relative to optic nerve and solitary orbital schwannomas.

## Introduction

Schwannomas are primary nervous system benign tumors arising from Schwann cells, which act normally to myelinate neurons peripherally beginning at the Obersteiner-Redlich zone, the transition from the central to the peripheral nervous system. These tumors are typically slow growing, encapsulated, and usually become symptomatic as a result of a mass effect on nearby structures. Intracranially, schwannomas account for up to 10% of primary tumors with the eighth cranial nerve (CNVIII) most commonly involved followed by the trigeminal nerve roots.

While CNV and VIII are by far the two most commonly affected cranial nerves, origination from the oculomotor, trochlear, or abducens nerves is certainly feasible given their inclusion as part of the peripheral nervous system. Solitary intraorbital schwannomas are rare lesions, accounting for up to 6.5% of orbital neoplasms [1].^ ^Clinical presentation ranges from insidious proptosis, visual field loss, retro-orbital pain and headaches, to in rare cases blindness. Because the optic nerve is myelinated by central nervous system oligodendrocytes rather than Schwann cells, it is not surprising optic schwannomas are exceedingly rare lesions with, to our knowledge, only five previously reported cases [2-5].

Herein, we discuss a rare case of histopathologically confirmed optic schwannoma and review the available literature.

## Case presentation

**Figure 1:**
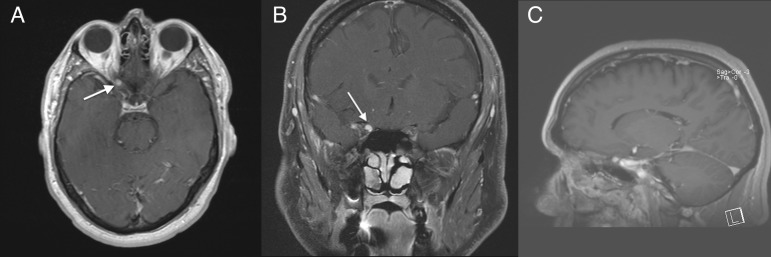
Preoperative T1-weighted MRI with contrast Axial view showing a small enhancing lesion along the optic nerve at the orbital apex pointed out by the white arrow (A). Coronal (B) and sagittal (C) views again showing the enhancing orbital mass.

The patient is a 46-year-old male who presented to the neurosurgery clinic with a one-year history of progressive right eye blurry vision, which he first noticed while serving as an umpire in a little league baseball game. He denied any headaches, although he had recently developed a painless pressure sensation behind the right eye. He had no contributory medical history (e.g. endocrinologic, vascular) to explain an optic mononeuritis, and there was no personal or family history of neurofibromatosis. An evaluation by neuro-ophthalmology showed severe degradation of the right eye visual fields with sparing only of the inferior medial field. Right optic atrophy was also appreciated on examination. Physical examination in the neurosurgery clinic was unremarkable for any focal neurological findings, and both of his pupils were equal, round, and reactive to light and accommodation. A T1-weighted MRI with contrast demonstrated a 4 x 5 mm homogeneously enhancing mass at the right orbital apex within the bony canal and along the medial aspect of the right optic nerve sheath with resultant mass effect on the right optic nerve (Figure 1).

The differential diagnosis at this point was optic nerve meningioma versus optic nerve glioma. Optic nerve signal was also consistent with neuritis on the right. After discussion of the operative plan in the clinic, the patient agreed to undergo endoscopic endonasal surgery for bony and dural decompression with resection of the tumor if it was felt to be amenable intraoperatively. Informed patient consent was obtained prior to treatment.

The neurosurgery team worked with otorhinolaryngology (ENT) for the skull base approach. The right-sided ethmoid air cells were opened to expose the medial orbital wall. A posterior septostomy was performed on the right, and the face of the sella was widely opened to expose the tuberculum sellae and planum sphenoidale superiorly. We removed the most distal portions of the lamina papyracea on the right. The right-sided optic canal and carotid prominence beneath were clearly visible. Image guidance with CT and MRI with contrast confirmed our location within these landmarks. Additional bone was removed from the proximal orbital apex and along the optic canal with a high-speed, diamond-tipped drill and irrigation. In this manner, we exposed the dura of the optic canal from where it opened into the orbital apex back to its intersection with the tuberculum sella. This portion of the procedure constituted the bony decompression. We then directly inspected the exposed dura and used neuro-navigation as well as visual landmarks to determine the location of the medial optic canal lesion. Directly over this, we made a linear incision in the dura and immediately encountered tannish-gray tumor material. It was somewhat tough in consistency and firmly attached to the optic nerve. At this point, the patient’s wishes to conserve vision in the right eye if possible was heavily taken into consideration, and given the previously mentioned difficult characteristics of the tumor, it was subtotally resected. Nevertheless, some of the central portions were acutely decompressed and samples were sent for permanent pathology. Although no CSF leak was frankly noted, there was concern the patient could develop such a leak in the future because of the small opening that was made in the dura. Therefore, we performed a direct repair using a free nasoseptal graft.

Following surgery, the patient did well with no apparent complications and was discharged on postoperative day two. The final permanent pathology specimens revealed a classic palisading pattern of tumor nuclei typical of schwannoma. 

**Figure 2:**
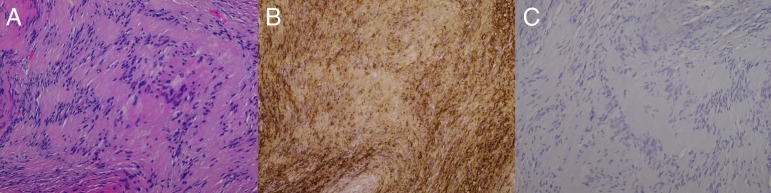
Pathology slides demonstrating a diagnosis of optic nerve schwannoma Hematoxylin and eosin slide at 20x showing the classic palisading pattern of schwannoma (A). Positive S100 stain (B) and negative EMA (endothelial membrane antigen) stain (C) both at 20x.

The specimen also stained positive for S100 and was negative for EMA (endothelial membrane antigen), thus confirming the diagnosis of schwannoma (Figure 2).

**Figure 3:**
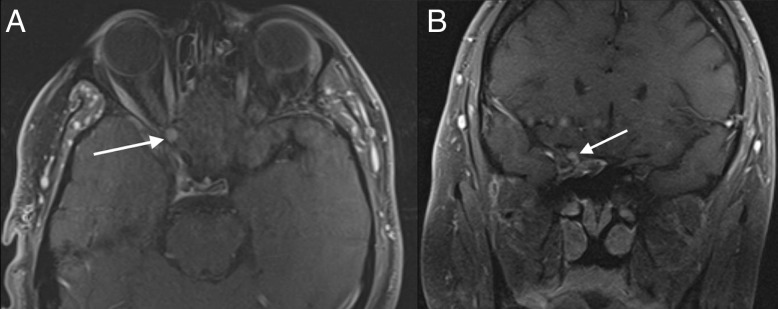
Postoperative T1-weighted MRI with contrast Axial view demonstrating a roughly stable enhancing schwannoma following decompression and biopsy as pointed out by the white arrow (A). Coronal view with the tumor again highlighted by the white arrow (B).

The patient was seen in clinic three months after his procedure and endorsed subjective improvement in his right eye superior quadrants; however, his preoperative visual deficits would return. Postoperative MRI at this time showed an approximately stable lesion compared to preoperatively (Figure 3).

He was instructed to see the neuro-ophthalmology and radiation oncology services for a new baseline examination and adjuvant radiotherapy versus observation of expected growth of the residual tumor. The patient chose not to follow up with ophthalmology. He ultimately completed a course of external beam radiation therapy (EBRT) for a total of 50.4 Gy in 28 fractions with IMRT to reduce the dose to the optic nerve and other critical intraorbital structures. He successfully completed this regimen without any new visual deficits nor complaints and is scheduled for annual MRIs and follow-up with neurosurgery or sooner if he experiences an interim visual decline.

Informed patient consent was obtained for reporting this case in the neurosurgical literature.

## Discussion

After a comprehensive review of the English literature, there have only been five previously reported cases of an optic schwannoma. To our knowledge, this was the first such case that was resected via an endoscopic endonasal approach. Additionally, we provide the only encounter of a schwannoma that arises from the optic nerve itself rather than the nerve sheath.

A recent study examining orbital schwannomas over a period of 18 years at one institution (49 total cases) demonstrated over 60% involvement of the superior orbit, which indicates the majority of these neoplasms probably arise from the orbital segment of the trigeminal nerve [1]. Given the inherent anatomy of the optic nerve, whose neuronal cells originate in the lateral geniculate bodies and are thus myelinated by oligodendrocytes, CNII schwannomas are very rare tumors and could easily be radiographically mistaken for more common lesions, such as optic meningioma, hemangioma, or glioma. On the basis of this strict anatomical knowledge, optic schwannomas should technically never develop, although their existence is thought to possibly be due to origination from small sympathetic nerves innervating the vasculature of the optic nerve and its sheath [2]. Because this particular tumor was seen arising more centrally within the optic nerve, we postulate its origin is from peripheral sympathetic nerves innervating the central retinal artery. Another less likely alternative is that the optic nerve occasionally contains aberrant Schwann cells and are no less subject to neoplastic transformation. However, several well-established studies have examined the source and pattern of myelination along the optic nerve, indicating that oligodendrocytes indeed myelinate the optic nerves in a proximal to distal progression [6-7].

An endoscopic endonasal approach (EEA) for orbital tumors provides adequate exposure of the medial orbital wall and orbital apex with minimal bleeding and lower complication rates [8-9]. Various other pathologies, such as cavernous hemangiomas, fibrous dysplasias, and squamous cell carcinomas, have been successfully resected with EEA in this location with favorable intraoperative exposure and postoperative results [10]. Because the tumor rested medially within the orbital apex, a transcranial approach was felt to have been less effective. We chose EEA, and resection and exposure throughout the case were more than adequate. A more invasive transcranial or transorbital approach, therefore, would not necessarily have allowed us a better opportunity to perform a gross total resection, which may not have been possible anyway given its tough consistency and continuity with the optic nerve.

Considering the extreme rarity of optic nerve schwannomas, the feasibility of adjunctive radiotherapy must be evaluated relative to previous data on conventional intracranial schwannomas and other types of intraorbital tumors. Fortunately, complication rates after radiotherapy for solitary orbital lesions is low. In cases of tumors abutting the optic nerve, radiotherapy has shown to be a safe adjunctive therapy with as few as 0% of patients suffering progression of visual deficits while almost 50% experienced some degree of improvement in their preoperative visual acuity and/or visual field deficits [11]. The patient expressed at the onset of treatment that salvaging vision in the right eye was a priority; therefore, the decision was made not to subject the optic nerve to radiosurgery. With this in mind, it was decided the patient could safely tolerate adjunctive radiotherapy and was scheduled to undergo focused EBRT. 

## Conclusions

Optic schwannomas remain extremely rare lesions, and the most effective treatment is based on that of more conventionally located lesions. This is only the sixth known reported case of an optic nerve schwannoma and the first that has been resected endoscopically. Despite subtotal resection in this case, EEA is a viable option for effectively treating inferomedial orbital apex tumors with adjunctive radiation. Not unlike other tumors in a similar location, optic schwannomas can be appropriately treated with endoscopy and adjuvant EBRT.
